# An investigation into the psychometric properties of the Hospital Anxiety and Depression Scale in patients with breast cancer

**DOI:** 10.1186/1477-7525-3-41

**Published:** 2005-07-14

**Authors:** Jacqui Rodgers, Colin R Martin, Rachel C Morse, Kate Kendell, Mark Verrill

**Affiliations:** 1 School of Neurology, Neurobiology and Psychiatry, Faculty of Medical Sciences, University of Newcastle, Newcastle upon Tyne, Tyne and Wear, NE17RU, UK; 2 The Nethersole School of Nursing, Faculty of Medicine, The Chinese University of Hong Kong, Esther Lee Building, Chung Chi College, Shatin, New Territories, Hong Kong, China; 3 Northern Centre for Cancer Treatment, Newcastle General Hospital, Newcastle upon Tyne, UK

## Abstract

**Background:**

To determine the psychometric properties of the Hospital Anxiety and Depression Scale (HADS) in patients with breast cancer and determine the suitability of the instrument for use with this clinical group.

**Methods:**

A cross-sectional design was used. The study used a pooled data set from three breast cancer clinical groups. The dependent variables were HADS anxiety and depression sub-scale scores. Exploratory and confirmatory factor analyses were conducted on the HADS to determine its psychometric properties in 110 patients with breast cancer. Seven models were tested to determine model fit to the data.

**Results:**

Both factor analysis methods indicated that three-factor models provided a better fit to the data compared to two-factor (anxiety and depression) models for breast cancer patients. Clark and Watson's three factor tripartite and three factor hierarchical models provided the best fit.

**Conclusion:**

The underlying factor structure of the HADS in breast cancer patients comprises three distinct, but correlated factors, negative affectivity, autonomic anxiety and anhedonic depression. The clinical utility of the HADS in screening for anxiety and depression in breast cancer patients may be enhanced by using a modified scoring procedure based on a three-factor model of psychological distress. This proposed alternate scoring method involving regressing autonomic anxiety and anhedonic depression factors onto the third factor (negative affectivity) requires further investigation in order to establish its efficacy.

## Background

A diagnosis of breast cancer is often accompanied by a significant and profound experience of psychological distress, the most commonly presenting symptoms being those of anxiety and depression [1]. Indeed, prevalence rates of clinically relevant levels of anxiety and depression in cancer patients have been estimated to be up to 45% [2-4]. It has been observed that psychological symptoms often decrease over time, further it has also been observed in the clinical presentation of breast cancer that up to 30% of these patients will continue to experience clinically relevant levels of anxiety and depression at follow-up [5].

The role of psychological variables, particularly those of anxiety and depression in disease progression and clinical outcome has received attention from the research community. For example, Walker et al. [6] found in a study of women with advanced breast cancer that anxiety and depression, as assessed by self-report measure, were significant predictors of the patients' response to chemotherapy in terms of clinical and pathological outcomes. Importantly, Walker and colleagues [6] identified that anxiety and depression were independent predictors of outcome, and therefore recommended that psychological factors need to be assessed and evaluated within the overall context of treatment.

The predictive account of the relevance of psychological factors is further supported by the findings of other studies. Hopwood et al. [7], found that high levels of anxiety and depression were associated with higher mortality rates in cancer patients. Ratcliffe et al. [8], found that high levels of depression were associated with higher mortality rates in patients with Hodgkin's disease and non-Hodgkin's Lymphoma.

Given the relevance of anxiety and depression to clinical outcome in individuals with a diagnosis of cancer, techniques and tools that reliably and consistently measure these important psychological dimensions would be welcomed within the therapeutic assessment and monitoring battery. Indeed, the need for application of psychometrically robust affective assessment tools to the clinical oncology setting is pressing due to inadequate training of non-specialist clinicians and nurses in recognising and screening for symptoms of psychological distress [9]. This is particularly important given the possible prognostic advantages offered by effectively identifying those individuals who may be anxious and depressed following diagnosis and treatment and then targeting specific interventions at these patients to reduce psychological sequelae [6].

In summary, there is convincing clinical evidence to suggest that a psychometrically robust, accurate, easily administered and patient acceptable affective state assessment tool could be of great benefit in assessing levels of anxiety and depression in patients with cancer.

The Hospital Anxiety and Depression Scale (HADS) [10] is a widely used self-report instrument designed as a brief assessment tool of the distinct dimensions of anxiety and depression in non-psychiatric populations [11,12]. It is a 14-item questionnaire that consists of two sub-scales of seven items designed to measure levels both of anxiety and depression. The ease, speed and patient acceptability of the HADS has led to it being applied to a wide variety of clinical populations where significant anxiety and depression may co-exist with the manifestation of physical illness [6,13-21].

The HADS has also been used widely in the clinical oncology setting as a screening and research tool [22-28]. Interestingly, conclusions drawn from investigations that have explored the utility of the HADS in the clinical oncology setting have yielded contradictory findings. A number of studies have suggested that the HADS reliably measures anxiety and depression in cancer patients [23,27,28] and should be adopted as a routine clinical tool for screening for psychological distress [29-31]. However, a number of other investigations in this area have suggested that the HADS may not be a suitable instrument to assess patients with cancer [24,32]. A general criticism of the HADS in cancer screening has been issues relating to the instruments poor sensitivity (ability to detect true cases) and specificity (ability to detect true non-cases) when tested against a 'gold standard', typically, a structured clinical interview [24,32].

However, a further issue concerns the method of scoring the HADS in relation to the HADS anxiety (HADS-A) and depression (HADS-D) sub-scales. A number of oncology studies [23,26,33-35] have suggested the HADS total score (all-14 items) should be used as a global measure of 'psychological distress'. This approach is against the recommendations of the original developers of the HADS [10] and this practice is further reproached in the HADS administration manual [36]. Razavi and colleagues [26] however, based their recommendation on a psychometrically robust rationale for using the HADS total score to assess cancer patients. Based on a number of psychometric criteria, including factor analysis and sensitivity/specificity criteria this study found just one single-factor emerged, identified as a single dimension of global psychological distress. This represents a good rationale for using the HADS as a unitary measure because it suggests that, in this population, the HADS could not discriminate between anxiety and depression.

However, Razavi et al.'s [26] findings of a single-dimension of global psychological distress have not been replicated in other studies examining cancer. Moorey et al. [37] found support for the bi-dimensional (anxiety and depression) underlying structure of the HADS in cancer patients. Interestingly, Moorey [37] did find some inconsistencies in their analysis with the HADS-A item 'I can sit at ease and feel relaxed' loading onto the HADS-D sub-scale. A further study examining anxiety and depression in patients with malignant melanoma [22] found the HADS to have an underlying three-factor structure. Lloyd-Williams [24] conducted an investigation into the utility of the HADS in terminally ill cancer patients and found a four-factor underlying dimensional structure.

Interestingly, a recent international consensus statement on depression and anxiety in oncology recommended the use of the HADS for screening cancer patients [38], however the recommendation was made on the explicit basis that the HADS 'assesses anxiety and depression as 2 dimensions scored separately' [38].

The factor inconsistencies observed in the HADS are not specific to studies involving cancer patients. Psychometric anomalies in the factor structure of the HADS have been observed in a diverse variety of clinical populations including depression [39], coronary heart disease [17], chronic fatigue syndrome [21], end-stage renal disease [16] and pregnancy [14]. A recent review [11] of studies that have investigated the underlying factor structure of the HADS found that nearly half reported factor structures inconsistent with the two-dimensional anxiety and depression model proposed by Zigmond and Snaith [11]. Despite the international use of the HADS in a vast multitude of clinical populations, the lack of systematic structural evaluation of the instrument in target clinical groups has been highlighted as an important psychometric concern.

Dunbar [40], conducted a confirmatory factor analysis of the HADS in a non-clinical population and found support for the three-factor tripartite model proposed by Clark & Watson [41]. This was a theoretically important observation since Clark & Watson's [41] three-factor tripartite model represents a development of the conceptualisation of anxiety and depression within a coherent and evidenced-based model. In addition their model is based upon a theoretically rich psychological account of anxiety and depression which is consistent with clinical observations of the disorders. Interestingly a number of recent psychometric investigations of the HADS have identified a three-factor underlying structure to the HADS in clinical populations [17,39].

Importantly, a recent investigation [21] into the psychometric properties of the HADS in individuals with chronic fatigue syndrome (CFS) tested Clark & Watson's three-factor tripartite model [41] and found it to provide a significantly better fit to the data than the bi-dimensional model proposed by Zigmond & Snaith [10]. McCue's [21] study extended the observations of Dunbar et al. [40] of support for the tripartite model to a clinical population. The relevance of this is that these findings suggest that a three-factor underlying structure to the HADS may have clinical implications since this model would be predicted by a coherent theoretical development, that of Clark & Watson [41], in the understanding of anxiety and depression within a clinical context. Interestingly, a number of studies have identified a third factor in the HADS using exploratory factor analysis, the researchers having then deciding to reject the third factor as meaningless and subsequently 'forcing' a two-factor solution [42,43]. It is likely that these researchers were not expecting to find a third factor since this would be inconsistent with Zigmond & Snaith's fundamental premise of bi-dimensionality of the HADS [10] and therefore chose to ignore the third factor in favour of an anticipated two-factor solution. A more recent study [20] used exploratory factor analysis and found an initial three-factor structure to the HADS in patients with end-stage renal disease. Martin and colleagues [20] then 'forced' a two-factor solution to their data and then compared the forced solution with the initial three-factor solution.

These investigators found the three-factor initial solution to be a much superior fitting underlying factor structure to the HADS compared to the 'forced' two-factor solution. It therefore seems possible that some researchers are in many instances rejecting an 'unexpected' three-factor structure in favour of the anticipated bi-dimensional structure. This is understandable in the absence of a credible theoretical perspective that would explain the manifestation of a three-factor dimensional structure to the HADS. Nonetheless, as has been established earlier, an alternative theoretical account does exist that would, in principle, predict a three-factor underlying structure to the HADS; the tripartite model of Clark & Watson [41].

However, it is important to note, that a departure from the bi-dimensional model of anxiety and depression supporting the HADS would suggest that the use of the HADS-A and HADS-D sub-scales for screening purposes would be seriously undermined since this is the fundamental rationale for using the HADS in clinical practice [38]. Conclusions drawn from HADS-A and HADS-D sub-scales would be unreliable, since the instrument would not in reality be measuring anxiety and depression and therefore clinical decision-making based on such scores would be fundamentally flawed [14,21]. See Table 1 for a summary of the models.

**Table 1:** Characteristics of each factor model tested

Model	No. Factors	Population	*n*	Extraction method	FLI1**	FLI2	FLI3
Zigmond et al(1983)	2	Medical	100	None	1,3,5,7,9,11,13	2,4,6,8,10,12,14	----
Moorey *et al. *(1991)	2	Cancer	568	PCA	1,3,5,9,11,13	2,4,6,7,8,10,12,14	-----
Dunbar et al (2000)	3	Non-clin	2,547^+^	CFA	1,5,7,11	2,4,6,7,8,10,12,14	3,9,13
Friedman *et al. *(2001)*	3	Depressed	2,669	PCA	1,7,11	2,4,6,8,10,12,14	3,5,9,13
Razavi *et al. *(1990)	1	Cancer	210	PCA	All items	---------	---------
Brandberg *et al. *(1992)	3	Cancer	273	PCA	3,5,9,13	2,4,6,8,10,12	1,7,11,14

*The three-factors are correlated in this model. ^+^Based on CFA of three independent samples of N = 894, 829 and 824, the total cohort in this study is 2,547.

^#^PCA: Principal Components Analysis; CFA: Confirmatory Factor Analysis. **FLI: Factor Loading Items. The HADS items loading on each model tested.

To date, no study has been conducted that has examined the factor structure of the HADS in cancer patients by comparing competing factor structures predicted by theoretical and evidenced-based accounts of psychological distress. There is a good rationale for pursuing this in cancer patients. Given that the HADS-A and HADS-D sub-scales have been demonstrated to have predictive outcome potential in the clinical oncology setting [6] establishing the best and most appropriate factor structure of the HADS in this group of clients may be a clinically useful way of improving the predictive capacity and reliability of the instrument [40]. The first step towards this goal is to establish the best factor structure and then undertake longitudinal research to establish the predictive value of that structure.

Most previous factor analyses of the HADS have used exploratory factor analysis (EFA) techniques, though there are a small number of recent and notable exceptions to this approach that have applied a more theoretically and clinically relevant methodology to data called confirmatory factor analysis [20,21,30,40].

This study seeks to determine the appropriateness of using the HADS as a two-dimensional instrument in women with breast cancer by examining the instrument's underlying factor structure using both EFA and CFA. The study will test the hypothesis that the HADS comprises a two-factor (anxiety and depression) underlying factor structure in women with breast cancer.

## Methods

### Design

The study used a cross-sectional design. To address the research questions exploratory factor analysis (EFA), confirmatory factor analysis (CFA) and reliability analysis methods were used using a pooled HADS data set from all participants. Relevant clinical details were also recorded.

### Statistical analysis

#### Reliability analysis

A reliability analysis of the HADS total all-items, and HADS anxiety (HADS-A) and HADS depression (HADS-D) sub-scales, was conducted to ensure that the measures satisfied the criteria for clinical and research purposes using the Cronbach coefficient alpha statistical procedure [44]. A Cronbach's alpha reliability statistic of 0.70 is considered as the minimum acceptable criterion of instrument internal reliability [45,46].

### Exploratory factor analysis

Exploratory factor analysis was performed on the full 14-item HADS. The criterion chosen to determine that an extracted factor accounted for a reasonably large proportion of the total variance was based on an eigenvalue greater than 1. A maximum likelihood factor extraction procedure was chosen on the basis that this approach is particularly useful in extracting psychologically meaningful factors [17,14,47]. A further advantage of using the maximum likelihood approach is that a chi-square statistic can be generated to determine if the number of extracted factors offers a statistically good fit to the model tested. An oblimin non-orthogonal factor rotation procedure was chosen [47] due to the possibility that extracted factors may be correlated. The arbitrary determination of a significant item factor loading was set at a coefficient level of 0.30 or greater, this level based on a rationale of maximising the possible number of items loading on emerging factors in order to generate a more complete psychological interpretation of the data set, this being a level consistent with investigators who have utilised exploratory factor analysis [14,17,48].

### Confirmatory factor analysis

Confirmatory factor analysis was conducted using the Analysis of Moment Structures (AMOS) version 4 statistical software package [49]. Seven models derived from clinical and empirical research were tested. These were Zigmond & Snaith's original two-factor model [10], Moorey et al.'s two-factor model [37], Razavi et al.'s single-factor model [26], Clark and Watson's three-factor tripartite model [41], Clark and Watson's three-factor hierarchical tripartite model [41] Friedman et al.'s three-factor correlated model [39] and Brandberg et al.'s three-factor correlated model [22].

For all models, independence of error terms was specified and the maximum likelihood method of estimation was used. Factors were allowed to be correlated where this was consistent with the particular factor model being tested. Multiple goodness of fit tests [50] were used to evaluate the seven models, these being the Comparative Fit Index (CFI) [51], the Akaike Information Criterion (AIC) [52], the Consistent Akaike Information Criterion (CAIC) [53] and the Root Mean Squared Error of Approximation (RMSEA). A CFI greater than 0.90 indicates a good fit to the data [54]. A RMSEA with values of less than 0.08 indicates a good fit to the data, while values greater than 0.10 suggest strongly that the model fit is unsatisfactory. The AIC and CAIC are useful fit indices for allowing comparison between models [40]. The Chi-square goodness of fit test was also used to allow models to be compared and to determine the acceptability of model fit. A statistically significant χ^2 ^indicates a proportion of the variance in the model remains unexplained by the model tested [50].

### Comparison with normative data

Comparison with the most contemporary normative HADS data in breast cancer patients [55] was conducted using the one-sample t-test.

### Procedure

An information sheet and consent form was posted to patients approximately three weeks prior to their routine clinic follow-up. Participants were either seen at home or at clinic by one of the researchers (RM) and completed a pack of questionnaires including the HADS. Participants also completed a short neurocognitive test battery. The study took 45 minutes to complete.

### Participants

110 women who had undergone adjuvant treatment for breast cancer, and were at least 6 months post-chemotherapy, participated in the study. Patients with a history of major psychiatric illness were excluded. Women were recruited from three treatment groups: chemotherapy alone, hormonal therapy alone, and a combination of chemotherapy and hormonal therapy.

Socio-demographic and treatment characteristics of the participant groups are presented in Table 2. A significant group effect of age was observed, F_(2,107) _= 3.09, p = 0.05, with women in the hormone therapy alone group being significantly older than women in the chemotherapy alone group (Bonferroni post-hoc test, p = 0.04). No other statistically significant differences were observed between groups, all further group comparisons of socio-demographic, baseline treatment data and HADS-A and HADS-D scores being conducted using analysis of co-variance (ANCOVA) controlling for age.

**Table 2:** Demographic and clinical data mean scores/levels with standard deviations in parentheses and accompanying F and p values of group comparisons.

	Group type				
	
Variable	Chemotherapy alone	Chemotherapy and hormone	Hormone alone	*F*	*p*
HADS-A	7.33 (3.99)	6.48 (3.96)	7.35 (4.90)	0.08*	0.92
HADS-D	3.11 (3.73)	2.90 (2.30)	4.35 (3.35)	1.95*	0.15
Length of time since treatment ended	2.49 (2.09)	2.53 (1.56)	1.83 (1.43)	1.69*	0.19
Townsend index of deprivation	-0.78 (2.76)	-0.94 (3.10)	0.37 (3.46)	1.20*	0.30
Age	52.52 (8.20)	55.24 (6.86)	57.95 (9.06)	3.09^#^	0.05

*Analysis of co-variance (ANCOVA) controlling for age, F_(2,106) _degrees of freedom.

^#^Analysis of variance (ANOVA), F_(2,107) _degrees of freedom.

The data was drawn from a larger study exploring neurocognitive and behavioural outcomes following breast cancer treatment. Ethical approval was obtained from Newcastle and North Tyneside Health Authority Joint Ethics Committee. Participants were recruited through the Northern Centre for Cancer Treatment and the Royal Victoria Infirmary, Newcastle upon Tyne, UK. Written informed consent was obtained from all participants prior to the commencement of the study.

## Results

The mean scores of participant's ratings on the HADS-A were 7.43 (SD 4.14) and HADS-D was 3.25 (SD 2.97). Using Snaith & Zigmond's interpretation of HADS-A and HADS-D scores of 8 or over, 51 participants (46.4%) demonstrated possible clinically relevant levels of anxiety and 11 patients (10.0%) possible clinically relevant levels of depression [10]. Adopting Snaith & Zigmond's higher threshold for sensitivity of HADS-A and HADS-D scores of 11 or over, 24 participants (21.8%) demonstrated probable clinically relevant levels of anxiety and 3 participants (2.7%) probable clinically relevant levels of depression [36].

### Reliability analysis

Calculated Cronbach's alpha of the HADS (all 14 items), HADS-A and HADS-D sub-scales was 0.85, 0.79 and 0.87 respectively, exceeding Kline's criterion for acceptable instrument internal reliability [45].

### Comparison with normative data

No statistically significant differences were observed between HADS-A (t_(109) _= 0.18, p = 0.85) and HADS-D (t_(109) _= 0.16, p = 0.87) mean scores of the current study compared to those of Osborne et al. [55].

### Exploratory factor analysis

The Kaiser-Meyer-Olkin (KMO) measure of sampling adequacy and the Bartlett Test of Sphericity (BTS) were conducted on the data prior to factor extraction to ensure that the characteristics of the data set were suitable for the factor analysis to be conducted. KMO analysis yielded an index of 0.86, and in concert with a highly significant BTS, χ^2^_(df = 91) _= 635.36, p < 0.001, confirmed that the data distribution satisfied the psychometric criteria for the factor analysis to be performed. Following factor extraction and oblimin rotation, three factors with eigenvalues greater than 1 emerged from analysis of the complete HADS and accumulatively accounted for 59.82% of the total variance. The factor loadings of the individual HADS items in relation to the three-factor solution are reproduced in Table 2.

Factor scores on each extracted factor for each participant were calculated using regression. In contrast with the Bartlett and Anderson-Rubin methods of factor score calculation, the regression method was chosen since this technique does not assume the extracted factors are orthogonal and also minimises any sum of squares discrepancies between true and estimated factors over individuals. Factor one proved to be highly statistically significantly, but negatively correlated with factor two, r = -0.48, p < 0.001. Factor one was significantly positively correlated with factor three, r = 0.45, p < 0.001. Factor two was observed to be highly statistically and negatively correlated with factor three, r = -0.63, p < 0.001. The chi-square goodness of fit test, χ^2^_(df = 52) _= 57.18, p = 0.29, was not statistically significant suggesting that the three-factor solution extracted provided a good fit to the data. A forced two-factor solution was then specified, however, the emergent factor solution failed to provide a good fit to the data, χ^2^_(df = 64) _= 85.62, p = 0.04. The forced two-factor solution accounted for only 45.08% of the total variance.

### Confirmatory factor analysis

**Figure 1:**
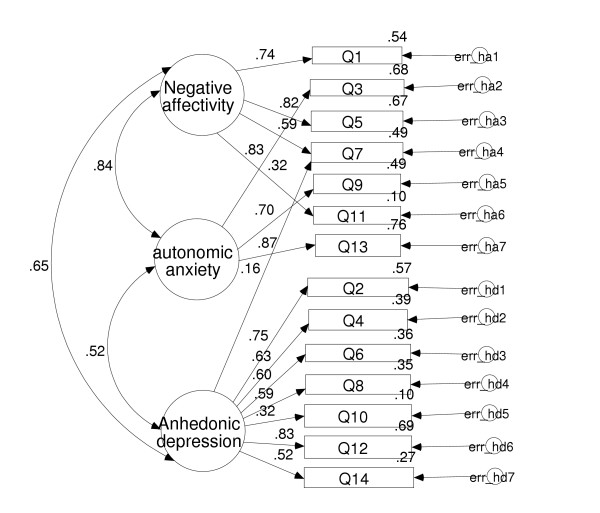
**Clark & Watson's (1991) Tripartite model applied to HADS data. **Note: Figures represent standardised parameter estimates.

The factor models tested and accompanying fit indices are shown in Table 3. The χ^2 ^goodness of fit analyses for all models were statistically significant (p < 0.05) indicating a proportion of the variance was unexplained by each model. Examination of the fit indices for each model revealed that the best fit to the data is Clark and Watson's [41] three-factor tripartite model, their being little difference between correlated and hierarchically correlated versions of the model (Figure 1). The second closest fit to the data was provided by Friedman et al.'s three factor model [39]. The third closest fit to the data was found to be Brandberg et al.'s [22] three-factor correlated model. Zigmond and Snaith's original two-factor model [10] offered the fourth best fit to the data, while the two-factor model of Moorey et al. [37] provided the fifth best fit. The worst fit to the data was furnished by the single factor model of Razavi et al. [26](Table 4).

**Table 3:** Factor loadings of HAD Scale items following maximum likelihood factor extraction with oblimin rotation

**HAD Scale item**	**Factor 1**	**Factor 2**	**Factor 3**
***Anxiety sub-scale***			
(1) I feel tense or wound up	0.17	**-0.30**	**0.45**
(3) I get a sort of frightened feeling as if something awful is about to happen	0.16	**-0.80**	-0.08
(5) Worrying thoughts go through my mind	0.24	**-0.55**	0.16
(7) I can sit at ease and feel relaxed	0.26	-0.10	**0.61**
(9) I get a sort of frightened feeling like 'butterflies' in the stomach	-0.18	**-0.79**	0.04
(11) I feel restless as if I have to be on the move	-0.06	0.01	**0.53**
(13) I get sudden feelings of panic	0.03	**-0.82**	0.04
***Depression sub-scale***			

(2) I still enjoy the things I used to enjoy	**0.72**	-0.04	-0.02
(4) I can laugh and see the funny side of things	**0.50**	-0.11	0.12
(6) I feel cheerful	**0.45**	-0.15	0.15
(8) I feel as if I am slowed down	**0.56**	0.07	0.18
(10) I have lost interest in my appearance	**0.35**	-0.01	-0.05
(12) I look forward with enjoyment to things	**0.88**	-0.08	-0.11
(14) I can enjoy a good book or TV programme	**0.58**	0.07	0.01

*Bold indicates that item loading on a factor is 0.30 or above

**Table 4:** Factor structure of the HADS determined by testing the fit of models derived from factor analysis.

**Model**	χ^2 ^**df**	***p***	**RMSEA**	**CFI**	**CAIC**	**AIC**
Zigmond and Snaith two-factor	121.77 (76)	0.001	0.07	0.92	287.08	179.77
Moorey *et al*. two-factor	132.16 (76)	<0.001	0.08	0.90	297.47	190.16
Friedman three-factor correlated	101.79 (74)	0.018	0.06	0.95	278.50	163.79
Dunbar *et al*. three-factor tripartite	**96.16 (73)**	**0.036**	**0.05**	**0.96**	**278.57**	**160.16**
Dunbar *et al*. three-factor hierarchical tripartite	**96.27 (73)**	**0.035**	**0.05**	**0.96**	**278.68**	**160.27**
Razavi single-factor	212.22 (77)	<0.001	0.13	0.77	371.83	268.22
Brandberg *et al*. three-factor	116.11 (74)	0.001	0.07	0.93	292.83	178.11

The best fit to the data is provided by the three-factor tripartite model and the three-factor hierarchical tripartite model of Clark & Watson (1991) based on Dunbar *et al*. (2000).

## Discussion

This study has yielded interesting and clinically pertinent observations regarding the HADS in relation to psychological screening in women with breast cancer. The finding of relatively high levels of anxiety (mean = 7.43) and low levels of depression (mean = 3.25) is entirely consistent with the most recent investigation reporting HADS normative anxiety (mean = 7.50) and depression (mean = 3.30) data in a relatively large (N = 731) population of women with breast cancer [55]. This finding is suggestive that the HADS-A and HADS-D sub-scales appear to be pathology specific and sensitive.

Estimations of internal reliability revealed Cronbach's alpha's of the HADS (all items) and the HADS-A and HADS-D sub-scales to be all statistically acceptable, indeed, these observations being entirely consistent with previous research into the psychometric properties of this instrument (Bjelland et al., 2002). The HADS-A and HADS-D sub-scales were found to be positively and statistically significantly correlated, an observation that is again consistent with previous research [11]. Taken together, the consistency of HADS-A and HADS-D scores between this study and normative breast cancer HADS scores, the good internal reliability of HADS-A and HADS-D sub-scales and confirmation of the anticipated significant positive correlation between HADS-A and HADS-D sub-scales suggests that the HADS has achieved a number of the psychometric credentials required to confer it's acceptability as a reliable and valid screening tool of anxiety and depression for use in women with breast cancer.

However, the results of the EFA and CFA add a further dimension to the debate over the psychometric integrity of this instrument in this clinical population and, indeed, provide compelling evidence that the assumed bi-dimensional anxiety and depression underlying structure of the HADS should be reviewed, particularly in patients with breast cancer.

The EFA of the HADS revealed an initial three-factor underlying structure which provided a good fit to the data. When compared to a forced two-factor solution, the initial three-factor model provided a better fit to the data, the two-factor forced solution offering a statistically poor fit to the data. This is a clinically pertinent observation since not only does this finding reveal that the HADS does not measure two distinct dimensions of anxiety and depression in this population, it informs the growing evidence base which has increasingly suggested that the HADS is not a reliable measure of anxiety and depression when used within the context of a wide range of pathology [14,20-22,26,39,56].

Examination of individual item loadings is illuminating. It was observed that the HADS-A sub-scale items 1. 'I feel tense or wound up', 7. 'I can sit at ease and feel relaxed' and 11. 'I feel restless as if I have to be on the move' loaded on extracted factor 3. This separation of HADS-A items has been observed previously in factor analysis of cancer patient data.

Brandberg et al. [22], in a study of patients with malignant melanoma (skin cancer), found a three factor structure to the HADS and identified a 'restlessness' factor comprising items 1, 7, 11 and 14. Item 14. 'I can enjoy a good book or TV programme' was not found to load on to the 'restlessness' factor reported by Brandberg and colleagues [22] in the current study, though with this exception, the loading of HADS-A items on this 'restlessness' factor is identical. Items 3. 'I get a sort of frightened feeling as if something awful is about to happen', 5. 'Worrying thoughts go through my mind', 9. 'I get a sort of frightened feeling like 'butterflies' in the stomach' and 13. 'I get sudden feelings of panic' loaded onto extracted factor three. This observation, is consistent, indeed identical, with that factor extracted and observed by Brandberg et al. (1992) and termed 'anxiety'. Item 1. 'I feel tense or wound up' was observed to also load onto factor 2, however it should be emphasised that this item loads more heavily on extracted factor 3. All the HADS-D items loaded onto factor 1, this extracted factor being consistent with the depression sub-scale these items are designed to measure. The findings from the EFA would suggest that the HADS is comprised of three underlying factors, these being depression, anxiety and restlessness.

The CFA both supports the findings of the EFA and provides further evidence to support the notion that the HADS is comprised of an underlying three-factor structure in breast cancer patients. It should be noted that, though the χ^2 ^analysis of all models tested were statistically significant, indicating a significant proportion of the variance of the model tested to be unexplained in the data, it is readily acknowledged that trivial variations in the data can lead to significant χ^2 ^test results [57] and therefore the usefulness of the test within the realm of CFA is that it provides an index of comparatively how well a model fits the data. The three-factor models tested proved to provide better fits to the data than the two two-factor models tested on virtually all indices of model fit. The single factor model tested revealed the poorest fit to the data of all the models.

Clark & Watson's [41] three-factor tripartite and three-factor hierarchical tripartite models [40] provided the best fit to the data, examination of the RMSEA and CFI fit tests revealing that these three-factor models satisfied the criteria for a good fit to the data. Interestingly, the participant population in Dunbar et al.'s study [40] was drawn from a non-clinical population and the basis for the study was to test a strong contemporary theoretically-based account of anxiety and depression, that of Clark & Watson [41]. The second best fit to the data (Clark & Watson's model fit being virtually identical will be treated as a single best fit model) was provided by Friedman et al.'s three-factor model [39]. Friedman et al.'s study was an EFA on HADS data from a psychiatric population, individuals being treated for depressive disorder [39]. This finding gives an indication to the possibility that the three-factor best model fit observed in the current study may not, essentially be related to the presenting pathology, since breast cancer and depressive disorder represent two distinct and aetiologically unrelated clinical presentations, therefore the superior (compared to the competing two-factor models tested) three-factor model fit of Friedman's model [39] may, in fact, be tapping into the basic fundamental factor structure of the HADS.

This observation would be supported by the findings of the model fit of Brandberg's three-factor model [22], superior to that of the two-factor models. Observation of an underlying three-factor structure to the HADS has been observed in a number of other studies investigating a broad spectrum of clinical and non-clinical populations [16,20,21]. There have also been a number of instances in studies of psychiatric disorder where a three-factor underlying structure to the HADS has been initially observed and has then been dismissed by the authors in favour of the (presumably) expected two factor solution [42,43]. Arguably and retrospectively, these studies suggest further support for a three-factor underlying structure to the HADS.

Brandberg et al. [22] commented that, in spite of finding support for a three-factor underlying structure to the HADS, there was not a need for a revision of the instrument, rather, it was suggested that further studies of the instrument should be conducted. It is now over ten years since Brandberg and colleagues study [22] and the accumulating evidence base concerning the factor structure of the HADS raises credible clinical issues regarding the utility of this instrument across a range of pathologies. The findings from the CFA in the current study revealed that the two-factor models tested [10,37] offered a poorer fit to the data compared to the three-factor models. However, it should be stressed that examination of the RMSEA and CFI of both these two-factor models revealed that they offered an acceptable fit to the data. This is a noteworthy observation since other studies which have found support for the three-factor model of the HADS have found evidence that two-factor models offer a very poor fit to the data [20,21]. In summary, the CFA findings from the current study support a three-factor underlying factor structure to the HADS, though poorer fitting two-factor models still provide an acceptable, albeit less so, fit to the data.

Two questions remain, firstly what is the HADS measuring within the context of a three-factor model and secondly, should the HADS be continued to be used as a bi-dimensional screening tool for the detection of individuals experiencing anxiety and depression?

The best fit to the data was provided by Clark & Watson's tripartite and hierarchical tripartite three-factor models [41], there being very little difference between the models statistically establishing that both models are measuring fundamentally the same constructs. According to Clark & Watson's [41] formulation of anxiety and depression, the three factors observed in the HADS would represent distinct dimensions of negative affectivity, autonomic anxiety and anhedonic depression. These theoretically derived models have been shown to provide a best fit to the data in two previous research investigations that have focused on both a non-clinical populations [40], and a clinical population of individuals with chronic fatigue syndrome [21]. Furthermore, Crawford et. al [58] in a study evaluating the reliability ad validity of the Positive and Negative Affect Schedule (PANAS) and its relationship with other measures of depression and anxiety including the HADS have recently provided further support for tripartite theory of anxiety and depression.

It must be acknowledged that a number of limitations will inevitably apply to the current study. It must be noted that the sample size for the study was borderline for conducting SEM with AMOS but was adequate by a number of conventional criteria. One must also take into account the suggestion that differing methodologies used across studies to undertake factor analysis may account for the differences found, see Martin [58] for a full discussion of these issues. Additionally the low mean depression scores for the sample, whilst consistent with other studies with similar populations, might result in the presence of a floor effect, thus limiting the variance within the sample. This may have resulted for the fact that in order to avoid the short term acute sequelae associated with intensive treatment all participants were at least two years from treatment at the time of the investigation.

This study has extended the observations of Dunbar et al. [40] and McCue et al. [21] to a further population with distinct pathology. It has been suggested by Dunbar [40] that by using the hierarchical tripartite model, the autonomic anxiety and anhedonic depression factors would be of greater value in discriminating between anxiety and depression than simply using HADS anxiety and depression sub-scale scores. Brandberg et al. [22] noted that the HADS-D sub-scale was the most useful for clinical purposes, though the rationale was not stated, it seems plausible to assume that this was because of the 'split' HADS-A sub-scale observed in their factor analysis. This observation is entirely consistent with that of Dunbar [40] who suggests a convincing rationale why the HADS is not a highly discriminative instrument in some populations is because the HADS-A and HADS-D sub-scale scores are contaminated by overlap between the three underlying factors. A method of significantly increasing discriminability has been suggested by Dunbar et al., [40] involving regressing autonomic anxiety and anhedonic depression factors scores on to the negative affectivity (third factor) sub-scale scores.

A further study would be required to establish the efficacy and desirability of this approach since comparison of factor derived scores would need to be compared against a gold standard such as a formal structured clinical interview schedule. Using this approach receiver operating characteristic (ROC) curves could be calculated to evaluate any relative improvement of regressed autonomic anxiety and anhedonic depression scores compared to HADS-A and HADS-D scores.

The RMSEA and CFI statistics revealed that the two-factor models tested offered acceptable fits to the data, with Zigmond & Snaith's original two-factor formulation [10] offering a slightly better fit to the data to that of Moorey et al.'s modified two-factor model [37]. It is worthy of comment that Zigmond & Snaith's [10] model was superior to that of Moorey et al.'s model [37], in spite of the latter researchers using a clinical cohort comprised exclusively of cancer patients. This observation offers further support to conclusions drawn from the three-factor models tested that the underlying factor structure of the HADS is relatively stable and the impact of pathology on the factor structure of the instrument may be relatively minor. One of the central tenet for supporting using the HADS is that it is easy to use, this of course, includes scoring the instrument. Whether, any significant benefits in discriminability that may be identified in using the regressed scores as suggested by Dunbar et al. [40] may be off-set by an increase in sophistication in terms of calculating regressed factor scores in clinical practice.

Obviously, this is an area for future investigation, however it is worth noting that a wide variety of health professionals use the HADS in clinical practice on an everyday basis and it is these individuals who may feel reluctant or lack the time to calculate regressed scores for the HADS unless there is a large improvement to be found in the instruments accuracy by doing so. A simple scoring algorithm would be a fundamental requirement if the approach suggested by Dunbar and colleagues [40] was to move from the arena of academic and clinical research into the natural environment for the HADS, everyday clinical practice.

On balance, and incorporating the above limitations of ensuring that the HADS remains an easy to use clinical screening instrument, it is suggested that HADS remains a useful screening instrument in the clinical oncology environment and may be scored and interpreted in the recommended manner [10,36]. However, further clinical research work is recommended in this area to determine if scoring the instrument as a three-factor measure offers any worthwhile benefits in case detection that may offset a more complicated scoring procedure. No evidence at all was forthcoming to suggest that the HADS should be used as a one-dimensional model of global psychological distress, the single factor model providing a very poor fit to the data. Based on this observation it is suggested that a total HADS score should not be used in this clinical context.

## Conclusion

In conclusion, a compromise is suggested based on the clinical research observations of the current study and the clinical context of everyday professional practice where the HADS is used as a screening instrument of choice. The HADS was found to have an underlying three-factor structure in breast cancer patients. The possibility that improved accuracy in case detection may be found by using a three factor model to score the HADS is balanced by a potential decrease in the ease of use of the instrument because a more complex scoring system will be required.

This issue can be settled by future research in this area to determine the magnitude of any worthwhile clinical gains in scoring the HADS as a three-factor instrument. Currently however, it is suggested that the HADS can be continued to be used and scored in the traditional way, since the two-factor models tested still provided an acceptable fit to the data. However, it is recommended that for screening purposes with breast cancer patients, verification of borderline level scores should be established by a structured diagnostic clinical interview. Those using the HADS in clinical practice may also wish to consider using further measures of negative affectivity and autonomic anxiety, since these are currently poorly represented in the HADS. The possibility that the HADS, or a derivative of the HADS, may be more usefully developed as a three-dimensional rather than bi-dimensional tool consistent with advances in psychological models of anxiety and depression [41] should not be ruled out.

## Authors' contributions

JR conceived of the study, participated in the design of the study, assisted in the analysis of the data and drafting of the manuscript. CM participated in the design of the study and performed the statistical analysis and drafted the manuscript. RM collected data, and contributed to the statistical analysis and interpretation of the results and the drafting of the manuscript. KK – participated in the conception and design of the study and the drafting of the manuscript. MV – provided access to participants, aided with the design of the study and participated in drafting the manuscript. All authors read and approved the final manuscript
